# How Did the Spider Cross the River? Behavioral Adaptations for River-Bridging Webs in *Caerostris darwini* (Araneae: Araneidae)

**DOI:** 10.1371/journal.pone.0026847

**Published:** 2011-10-26

**Authors:** Matjaž Gregorič, Ingi Agnarsson, Todd A. Blackledge, Matjaž Kuntner

**Affiliations:** 1 Scientific Research Centre, Institute of Biology, Slovenian Academy of Sciences and Arts, Ljubljana, Slovenia; 2 Department of Biology, University of Puerto Rico, San Juan, Puerto Rico, United States of America; 3 Integrated Bioscience Program, Department of Biology, University of Akron, Akron, Ohio, United States of America; 4 Department of Entomology, National Museum of Natural History, Smithsonian Institution, Washington, DC, United States of America; 5 College of Life Sciences, Hubei University, Wuhan, Hubei, China; Arizona State University, United States of America

## Abstract

**Background:**

Interspecific coevolution is well described, but we know significantly less about how multiple traits coevolve within a species, particularly between behavioral traits and biomechanical properties of animals' “extended phenotypes”. In orb weaving spiders, coevolution of spider behavior with ecological and physical traits of their webs is expected. Darwin's bark spider (*Caerostris darwini*) bridges large water bodies, building the largest known orb webs utilizing the toughest known silk. Here, we examine *C. darwini* web building behaviors to establish how bridge lines are formed over water. We also test the prediction that this spider's unique web ecology and architecture coevolved with new web building behaviors.

**Methodology:**

We observed *C. darwini* in its natural habitat and filmed web building. We observed 90 web building events, and compared web building behaviors to other species of orb web spiders.

**Conclusions:**

*Caerostris darwini* uses a unique set of behaviors, some unknown in other spiders, to construct its enormous webs. First, the spiders release unusually large amounts of bridging silk into the air, which is then carried downwind, across the water body, establishing bridge lines. Second, the spiders perform almost no web site exploration. Third, they construct the orb capture area below the initial bridge line. In contrast to all known orb-weavers, the web hub is therefore not part of the initial bridge line but is instead built *de novo*. Fourth, the orb contains two types of radial threads, with those in the upper half of the web doubled. These unique behaviors result in a giant, yet rather simplified web. Our results continue to build evidence for the coevolution of behavioral (web building), ecological (web microhabitat) and biomaterial (silk biomechanics) traits that combined allow *C. darwini* to occupy a unique niche among spiders.

## Introduction

Coevolution, change of one trait triggered by shifts in a related trait [1], can occur at many hierarchical levels from amino acids to interspecific arms races [2]–[4]. While species coevolution is well documented, we lack a broad understanding of how multiple traits coevolve to enable resource use within a species. This particularly holds true for the potential coevolution of traits that lack obvious genetic linkage, such as ecological “extended phenotypic” (e.g. spider webs and their microhabitat), behavioral (e.g. web building behaviors), and biomechanical (e.g. intrinsic properties of silk) traits [5].

Spider webs are physical manifestations of web building behaviors and are built using some of the world's “highest performance” biomaterials – spider silks. Spider webs are thus ideal for studying coevolution between behaviors, ecology, and performance of biomaterials [6]–[8]. The orb web's evolutionary origin defines a single clade, Orbiculariae, a large and diverse group with more than 12.000 species [9]–[12]. Architectural evolution of orb webs through time has resulted in novel web types [9], [13], [14], such as the linyphiid sheetwebs and theridiid cobwebs [10], [15], [16], the deinopid casting web [17], as well as many modifications of the classical orb web [7], [9], [18]–[20]. Because spiders build orb webs using highly stereotypical behaviors that are evolutionarily conserved and phylogenetically informative [13], [20], the evolution of new web architectures are expected to coincide with novel behaviors.

The impressive range of web designs within the Orbiculariae represents adaptations to a large range of prey types in diverse habitats [7], [8]. Two major components in spider web evolution are the changes in quality (intrinsic material properties) of the different types of spider silk composing webs and the changes in behaviors associated with how those silks are assembled to produce the finished web (web building and architecture) [21], [22]. In particular, material properties of spider silk coevolve with web design among orb spiders, a coevolutionary pattern not clearly demonstrated in many other common biomaterials such as byssal threads, tendon and keratin [5]. However, the actual behaviors that orb web spiders use are largely unstudied in this context.

**Figure 1:**
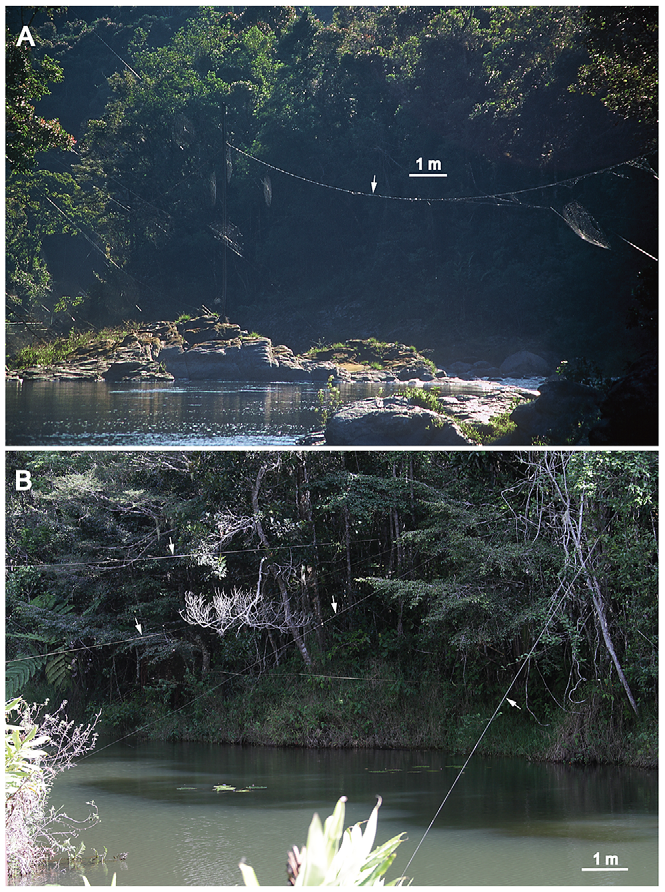
*Caerostris darwini* webs (white arrows indicate bridge lines) suspended above water in Andasibe Mantadia NP with whole orbs (A) and only bridge lines (B) visible.

Due to its amazing web architecture and silk toughness [23], the recently discovered Darwin's bark spider (*Caerostris darwini* Kuntner and Agnarsson 2010) is a promising system for studying the coevolution of behavioral traits with biomaterials during adaptation to new habitats. This species is endemic to Madagascar and is unique in building giant webs across streams, rivers and lakes. Some other spider species build smaller webs at the edges of waterways. However, no other spider builds webs that utilize the air column above large water bodies as habitat (Fig. 1) [7], [23]. The webs of *C. darwini* are made of silk combining strength and great elasticity such that it outperforms all other known spider silks, and even most synthetic fibers, in terms of toughness (work required to fracture the silk) [24]. Furthermore, capture areas of *C. darwini* webs regularly exceed 1 m in diameter and are suspended on bridge lines that often exceed 10 meters, while the largest capture areas reach almost 2 meters in diameter and are suspended on bridge lines up to 25 meters in length. These webs surpass even the gigantic *Nephila* webs, making *C. darwini* orb webs the largest known [23], [25], [26]. However, nothing is known about potential behavioral adaptations used to construct these giant webs in such unique microhabitats.

We thus pose several questions. First, bridging open space is a challenge for “typical” orb web spiders [18]. How then, do the enigmatic Darwin's bark spiders bridge such enormous distances across water? Second, did *C. darwini* evolve adaptations in web building behavior that accompany novel silk properties in achieving their unique webs? If so, did these behaviors evolve as modifications of existing orb web-weaving behaviors or are these behaviors evolutionary novelties? To address these questions, we designed a field study in eastern Madagascar and collected data on *C. darwini* web building and the site exploration behavior that precede web building [27].

## Results

**Figure 2:**
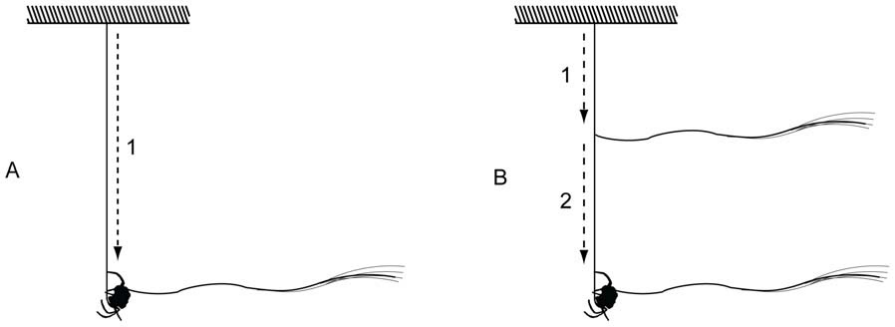
The typical (A) and the alternative (B) bridging behaviors in *C. darwini*.

We observed numerous *C. darwini* establishing bridges over open water bodies (Fig. 1) by descending on a dragline from their resting places in vegetation and releasing large amounts of silk into the wind (hereafter “bridging silk”; Fig. 2A). Bridging silk always constituted tens of silk threads that broadly exit the spinnerets and then formed into a single line after 24 seconds (median (ME), interquartile range (IQR)  = 18.75; N = 14; Video S1, S2). After the bridging silk eventually became entangled in vegetation or other substrates, typically on the other side of the water body, spiders (N = 19) started reeling in the silk, thus increasing its tension. If the attachment broke, the spiders reeled the loose silk up and consumed it, then continued attempting to establish bridge lines. If the attachment held, the spider crossed over the bridge line. When the spiders first crossed open spaces, they all (N = 19) cut and reeled the original bridging line as they laid a new one behind, as seen in other orb spiders (e.g. [27]–[29]). The spiders then reinforced the bridge line and both attachment points several times. To connect the bridge line with a third attachment point, all spiders (N = 32) gradually descended towards ground on a dragline while simultaneously releasing a new bridging silk thread into the air. The spider continued descending its dragline, until either successfully attaching the bridging silk to some distant substrate or reaching solid ground. We never observed connections to the water surface, but silk was instead always connected to vegetation sticking out of water or to shore vegetation. Spiders that contacted water crawled up the dragline thread to the original bridge line where they established a new dragline connection and then repeated the above mentioned behaviors until the spiders found solid surface for attachment. This apparent constraint on the placement of anchor lines implies that *C. darwini* webs typically could only be constructed close to the shore. However, this was not the case as many water bodies in these habitats were populated by semiaquatic plants that were used as substrate for web attachments. Also, webs were often constructed in the middle of water bodies, attached not only using long bridge lines but also long third anchor lines.

In a few cases, *C. darwini* departed from the usual bridging behavior by first attaching a bridging line on the dragline from which they were hanging, and then continuing bridging attempts with a second bridging line (Fig. 2B). However, these spiders never successfully completed such bridging threads.

**Figure 3:**
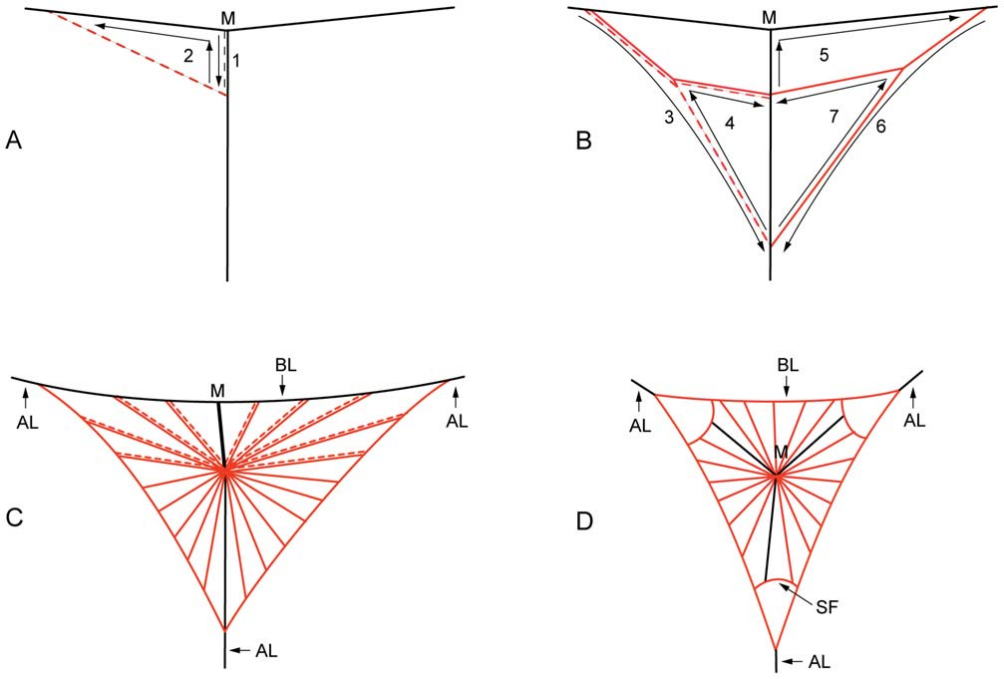
Web building in *C. darwini* (A-C) versus the “classical” araneid web (D), shown without spirals. AL. Anchor line. BL. Bridge line. M. Connection point of the initial bridge line and vertical anchor line. SF. Secondary frame. Numbered arrows show building sequence of web frame. A dashed and a solid line in “C” indicate doubled radii.

Up to this point, all spiders (N = 32) constructed a structure consisting of a more or less horizontal bridge line and a more or less vertical thread (Fig. 3A). This structure never resembled the textbook “Y” built by other orb spiders (e.g. [30], [31]), but rather a “T” (hereafter “T structure”). The junction of the two threads (hereafter “T junction”) never matched the proto-hub (hub of the future web), and the capture area was always built entirely below the bridge line (Fig. 3). The horizontal thread of the T structure was always converted into the bridge line and two horizontal anchor threads in the finished web, and the vertical thread was converted into two vertical radii and the lower anchor line. In contrast, other orb weavers build a Y shaped initial structure, where the three arms meet at the proto-hub and are converted into (replaced by) radii and anchor lines in the finished web so that the capture area is built around them (Fig. 3) [27], [31]–[34].

*Caerostris darwini* never built more than three anchor points (N = 32). Although some spiders showed certain levels of web site exploration by establishing up to three bridging silk attachments, we never observed exploration after establishing the T structure. Thus, the proto-orbs (primary frame, proto-hub and proto-radii, constructed together just prior to construction of the rest of the radii and spirals) were highly stereotypical, i.e. always consisted of the same arrangement of threads (Fig. 3B). To build a frame, all spiders walked down a part of the vertical thread of the T structure, laying a new silk thread behind, thus doubling this part of the vertical thread. In finished webs, the point where spiders attached the second vertical thread became the hub (Fig. 3A–C), and the doubled part of the vertical thread became a vertical radius. The spiders then built the orb web's side frames together with the first radii (Fig. 3B). We never observed secondary frame construction, which occurs in other orb weaving spiders when building radii (the secondary frame is thus connected to the threads of the primary frame; Fig. 3SF) [34], [35].

All observed spiders (N = 9) constructed single radii in the lower orb half (Fig. 3C). The spiders first laid a silk thread when moving from hub to frame, then cutting and reeling it using their third legs when returning to the hub, and simultaneously laying a new thread behind. Radii in the upper orb half were constructed as double radii, lacking the cut and reel of the previous thread. The silk remains of the cut and reeled threads in the lower orb were deposited at/near the hub, which sometimes appeared like rudimentary stabilimenta [36] in finished orbs. During radius construction, spiders reinforced the hub several times with additional loops of silk.

All spiders (N = 43) built the non-sticky spiral (NSS) from the hub to orb periphery, and the sticky spiral (SS) on the way back towards the hub. They attached the SS to every radius crossed and removed the NSS in the process. They determined the SS attachment point by tapping with the outer leg I. After finishing SS construction, spiders tested the web tension by shaking at the hub. We never observed hub destruction behavior and never observed building of ‘web decorations’ (stabilimenta), although the latter were occasionally found in webs in the field. Additionally, we observed one individual of an undescribed *Caerostris* species building radii, spirals and the hub the same way as *C. darwini*, and webs of all other encountered *Caerostris* species also lacked secondary frames.

The time *C. darwini* used to cross a water body (time from start of bridging behavior to reinforcement of future bridge) was 5–163 min (ME  = 52.5, IQR = 118.25; N = 7). The spiders then spent 6–43 min (ME  = 15, IQR = 13.13; N = 8) building the vertical anchor line, reinforcing the attachments and building the web frame. After that, the spiders used 3–9 min (ME  = 6.75, IQR = 2.15; N = 10) and 22–64 min (ME  = 42.5, IQR = 18.38; N = 6) to construct the radii and spirals, respectively. During web renewal, *C. darwini* (N = 29) completely removed and rebuilt the radii and SS, as well as frame threads outlining the capture area. They only reinforced the anchor lines, and thus both bridge lines as well as third anchor lines established across the water bodies are maintained long term.

## Discussion

*Cearostris darwini* uses a set of previously unknown behaviors to build orb webs in the air column above large water bodies. The spider produces unusually large amounts of bridging silk, almost completely lacks web site exploration behavior, has highly stereotypical proto-orb construction, builds the whole capture area below the initial bridge line, and constructs two types of radii in the same web. *Caerostris darwini* also anchors the web at only three points, and lacks both secondary web frames and hub modification. Other web building behaviors, such as spiral construction, are typical of other araneids [13], [20], [37]. We hypothesize that both the extreme mechanical properties of silk and the combination of web building behaviors in *C. darwini* represent adaptations to their novel environment.

Recent literature reports that orb spiders typically initiate web building by bridging using a single silk thread composed of minor ampullate silk, which is tightly interconnected with strands of aciniform silk [28], [38]–[40]. This behavior is also used by larger spiders to move to new web sites. In contrast, most small spiders disperse aerially (balloon) using a similar silk thread but with sail like terminus composed of numerous spread out silk strands, providing larger surface area [41]. However, our observations agree with older literature on bridging silk that suggest it also initially consists of numerous spread out silk strands [18], [35], [42], [43]. During ballooning, spiders typically climb to, and release silk from higher ground, while bridging behavior in spiders starts with a descent on a dragline. Our observations indicate that *C. darwini* does not differ from other orb weavers in the general structure of the bridge thread but rather in the quantity of threads attached to the main line (Video S1, S2). Such large amounts of silk are probably necessary to carry the bridge line over a sufficient distance to span large rivers and lakes, similar to dispersal via ballooning. Furthermore, the similarity between the bridging and ballooning behaviors of orb spiders suggest that the bridging behavior probably evolved from the ballooning behavior, the latter being known in almost all araneomorph spiders [6], [38].

*Caerostris darwini* bridging behavior is somewhat flexible. Several *C. darwini* individuals exhibited an alternative bridging behavior, attaching bridging lines while descending on their draglines and additionally releasing new bridging silk. Similar behavior is either facultative or predominant in other orb weavers [18], [42]. However, this alternative behavior was rare and never successful in our observations so that it likely plays only a minor role in bridging relatively short distances. As in other spiders [29], prior to crawling on the new bridge line, *C. darwini* reels the newly attached bridging silk, thus increasing the tension and testing the attachment strength. However, this is the first observation of spiders using bridging behavior to establish the third anchor lines (Fig. 3AL). Although this behavior might be present but simply not reported for other spider species, it would be more advantageous in spiders building over water where there are no or few attachment opportunities below the web, such as in *C. darwini*, compared to the majority of orb weavers who build over land.

According to the “refined gravity hypothesis” bridges sag under the weight of spiders and bridging to move between web sites could thus be less efficient in larger spiders that produce long bridges with more elastic silk [40], [44]. Ultimately, movement by larger orb spiders could be limited to short distances if their bridge lines sag too much. Our findings may contradict the refined gravity hypothesis as *C. darwini* are among the largest orb weavers and their silk is extremely elastic [5], [24], [26], yet they bridge larger distances than any other known orb spider. However, orb webs are suspended on bridge lines made of the unusually elastic major ampullate silk, while the initial bridging line when crossing open space is thought to consist of minor ampullate silk, whose mechanical properties are not yet known for *C. darwini*.

*Caerostris darwini* webs are relatively simple and this may relate to the webs' habitat. For example the web site exploratory behavior, as performed by most orb weavers and preceding web building *per se*, probably serves to avoid obstacles for the web's capture area [27], [32], [34]. These exploratory behaviors are not stereotypical as the environment is usually highly variable [27]. The resulting proto-orbs thus vary even within the same individual, and some components of the proto-orbs are not part of the finished webs [32], [34]. However, the air column above open water is typically obstacle free, and hence *C. darwini* need not perform additional exploration beyond the T-structure. We hypothesize that the open habitat above water led to the evolutionary loss of complex exploratory behaviors thereby resulting in the highly stereotypical and simplified proto-orb construction in *C. darwini*.

Uniform proto-orbs in *C. darwini* are always followed by suspension of the web on three anchor lines, the minimum necessary for a planar orb web. Searching for additional anchor points in the same plane would be uneconomical considering the scarcity of anchor points over water (e.g. vegetation) and the relative distance between shores. Webs of *C. darwini* also lack secondary frames, which most other orb weavers incorporate into their webs to lower the tension along radii [45]–[47]. Radii in a web as large as this may be under lower tension and therefore secondary frames may not be needed, but this remains to be tested. Other simplified features of *C. darwini* orb webs include few radii (15–30 [23], [25]), broad spiral mesh (5.9–30.5 [23], [25]) and the lack of hub destruction behavior [48]. After finishing spiral construction, *C. darwini* leave the hub intact, which while typical of species from other orb-weaving families, is unusual in araneids, most of which bite out and replace the hub silk [10], [13], [20], [49].

Perhaps the most striking differences between *C. darwini* webs and those of other known orb weavers are two features: i) the unique building of the whole capture area under the initial bridge line (Fig. 3) [30], [32]–[34], and ii) the combination of two types of radii in the same web – single radii in the lower and double radii in the upper orb web half. First, our results indicate that in *C. darwini*, bridging instead of web site exploration is the more energetically costly part of web building [34], here probably even intensified by large amounts of bridging silk used. We argue that retaining the long bridge in its entirety, as *C. darwini* does, is advantageous because both the silk and time investment, and thus energy investment in the functional bridge, is higher in an over-river habitat compared with terrestrial air columns with relatively shorter bridges. Other orb weavers typically modify and subsequently destroy and rebuild the initial bridge.

Second, *C. darwini* combines single and double radii in the same web, whereas most orb weavers only build single radii, except uloborids and nephilids which construct double radii throughout their webs [13], [20], [37]. However, a handful of other araneids also double their radii near the periphery of the web [50], [51], where the tension within a radius is higher [45], [46], [48]. Double radii in *C. darwini* could have several functions. First, in other orb weavers, radii in the upper half of orb webs are under higher tension [52]. As *C. darwini* build large webs with few radii, but also do not build secondary frames that reduce tension of long radii [45], [47], doubled radii may thus simply have the advantage to distribute force across more silk. Second, hub modification after orb construction is associated with adjusting tension in radii [48]. As *C. darwini* do not modify hubs after orb construction, adding another thread to radii in the upper half of the orb, perhaps pulled more/less tightly, might serve as a mechanism of adjusting tension. As at least some other *Caerostris* species also build simple webs containing two types of radii, their web structure might represent a preadaptation for building oversized webs across rivers and lakes, but may also not play a role in conquering this unique habitat at all.

Our results provide a strong evidence for the coevolution of behavioral web building traits with ecological traits such as web microhabitat, which in turn is linked to the exceptional material characteristics of silk in *C. darwini*. However, in the absence of a species level phylogeny, we cannot precisely pinpoint the exact origins of each of these traits. Nevertheless, the fact that some of *C. darwini* building behaviors, e.g. the simplified web and the building of two types of radii, are shared with at least some congeners, implies that these behaviors may have arisen at a deeper hierarchical level. Future research should focus on the precise order of evolutionary events in *Caerostris*. Therefore, we plan to integrate phylogenetic, taxonomic, behavioral and mechanical research of additional species of *Caerostris* into a coherent picture elucidating the fascinating web biology of these spiders.

## Materials and Methods

We documented web building behavior of *Caerostris darwini* females (adult or subadult) at several localities in Andasibe-Mantadia National Park (between S18.94760 E48.41972 and S18.79841 E48.42631 at roughly 960 m elevation), Toamasina Province, eastern Madagascar, between 24 February and 4 April 2010. In total, we observed 90 whole or partial web building events. We filmed and photographed selected behavioral sequences using camcorders (Sony DCR-SR87 HDD) and Canon SLR cameras (EOS 5D Mark II and EOS 7D).

Research, collecting, and export permits were obtained from The National Association for the Management of Protected Areas in Madagascar and Ministère de L'environnement, des Forêts et du Tourisme (permits Nu 087/08, Nu 088/08, and Nu 091N-EA04/MG08), through the Institute for the Conservation of Tropical Environments offices in Stony Brook and Antananarivo. Permits are on file with IA.

In 32 of 90 web building events, we started our observations at the beginning of web building. To do so, we at least partially destroyed *C. darwini* webs and then monitored them. To force the spiders to build a new bridge line, we sometimes (N = 19) destroyed the entire web including the bridge. In others (N = 13), we destroyed the capture area and all frame threads below the bridge, leaving the latter intact. We observed whole web building events in 18 of these 32 web building events. In the other 14, we had to terminate our observations prior to spiral construction, but did observe web building until the construction of the whole web frame and at least some radii. In 58 of all 90 web building events, we started our observations during spiral construction (N = 43) or radius construction (N = 15). Additionally, we sampled all radii of four webs on microscope glass slides to subsequently examine them under 1000x magnification, in order to confirm that all radii in the upper and lower orb web half are double and single stranded, respectively.

## Supporting Information

Video S1 ***C. darwini*** **using bridging silk.** Note the sail-like terminus of the bridging silk.
(MPG)

Video S2 ***C. darwini*** **using bridging silk.** Note the sail-like terminus of the bridging silk.
(MPG)
